# Posttraumatic stress disorder symptoms among trauma-exposed adolescents from low- and middle-income countries

**DOI:** 10.1186/s13034-021-00378-2

**Published:** 2021-06-05

**Authors:** Dusko Stupar, Dejan Stevanovic, Panos Vostanis, Olayinka Atilola, Paulo Moreira, Katarina Dodig-Curkovic, Tomislav Franic, Ana Doric, Nikolina Davidovic, Mohamad Avicenna, Isa Noor Multazam, Laura Nussbaum, Abdul Aziz Thabet, Dino Ubalde, Petar Petrov, Azra Deljkovic, Antonio Luis Monteiro, Adriana Ribas, Mirjana Jovanovic, Oliveira Joana, Rajna Knez

**Affiliations:** 1 Clinic for Neurology and Psychiatry for Children and Youth, Belgrade, Serbia; 2 School of Psychology, Leicester University, Leicester, UK; 3 Department of Behavioural Medicine, Lagos State University College of Medicine Ikeja, Lagos, Nigeria; 4 Lusíada University, Porto, Portugal; 5 CIPD, Porto, Portugal; 6 Medical Faculty Osijek, University Health Center Osijek, Osijek, Croatia; 7 Child and Adolescent Psychiatry, School of Medicine, University of Split, Split, Croatia; 8 Department of Psychology, Faculty of Humanities and Social Sciences, Rijeka, Croatia; 9 Faculty of Psychology, State Islamic University Syarif Hidayatullah, Jakarta, Indonesia; 10 Dr Soeharto Heerdjan Mental Hospital Jakarta, Jakarta, Indonesia; 11 Department of Child and Adolescent Psychiatry, University of Medicine and Pharmacy “Victor Babes”, Timisoara, Romania; 12 School of Public Health, Gaza Branch, Al Quds University, Jerusalem, Palestinian Territories Israel; 13 Department of Psychology, St. Dominic College of Asia, City of Bacoor, Bacoor, Philippines; 14 Department of Child and Adolescent Psychiatry, University Hospital St. Marina, Varna, Bulgaria; 15 Mental Health Center, Pljevlja, Montenegro; 16 Universidade Estacio de Sá in Rio de Janeiro, Rio de Janeiro, Brazil; 17 Federal University of Rio de Janeiro, Rio de Janeiro, Brazil; 18 Psychiatric Clinic, Clinical Center Kragujevac, Kragujevac, Serbia; 19 Lusíada University, Porto, Portugal; 20 Department of Pediatrics, Skaraborgs Hospital Skövde, Skövde, Sweden; 21 Department of Psychiatry and Neurochemistry, Institute of Neuroscience and Physiology, Sahlgrenska Academy, University of Gothenburg, Gothenburg, Sweden

**Keywords:** Traumatic events, Prevalence, Culture, PTSD-RI-5, UCLA PTSD index

## Abstract

**Background:**

Exposure to traumatic events in childhood is associated with the development and maintenance of various psychiatric disorders, but most frequently with posttraumatic stress disorder (PTSD). The aim of this study was to evaluate the types of traumatic events experienced and the presence and predictors of PTSD symptoms among adolescents from the general population from ten low- and middle-income countries (LMICs).

**Methods:**

Data were simultaneously collected from 3370 trauma-exposed adolescents (mean age = 15.41 [SD = 1.65] years, range 12–18; 1465 (43.5%) males and 1905 (56.5%) females) in Brazil, Bulgaria, Croatia, Indonesia, Montenegro, Nigeria, the Palestinian Territories, the Philippines, Romania, and Serbia, with Portugal, a high-income country, as a reference point. The UCLA PTSD Reaction Index for the DSM-5 (PTSD-RI-5) was used for the assessment of traumatic events and PTSD symptoms.

**Results:**

The most frequently reported traumatic events were death of a close person (69.7%), witnessing violence other than domestic (40.5%), being in a natural disaster (34.4%) and witnessing violent death or serious injury of a close person (33.9%). In total, 28.5% adolescents endorsed two to three DSM-5 PTSD criteria symptoms. The rates of adolescents with symptoms from all four DSM-5 criteria for PTSD were 6.2–8.1% in Indonesia, Serbia, Bulgaria, and Montenegro, and 9.2–10.5% in Philippines, Croatia and Brazil. From Portugal, 10.7% adolescents fall into this category, while 13.2% and 15.3% for the Palestinian Territories and Nigeria, respectively. A logistic regression model showed that younger age, experiencing war, being forced to have sex, and greater severity of symptoms (persistent avoidance, negative alterations in cognitions and mood, and alterations in arousal and reactivity) were significant predictors of fulfilling full PTSD criteria.

**Conclusions:**

Nearly every third adolescent living in LMICs might have some PTSD symptoms after experiencing a traumatic event, while nearly one in ten might have sufficient symptoms for full DSM-5 PTSD diagnosis. The findings can inform the generation of PTSD burden estimates, allocation of health resources, and designing and implementing psychosocial interventions for PTSD in LMICs.

## Background

Experiencing a traumatic event is common to all people across the globe, with up to 70% of adults reporting exposure to traumatic events [1]. Such extensive exposure also seems to be common in youth, since it was found that almost two-thirds of youth report life-time exposure to at least one traumatic event [2]. Exposure to traumatic events in childhood is associated with the development and maintenance of various psychiatric disorders, such as anxiety, depressive, somatic, but most frequently with posttraumatic stress disorder (PTSD; e.g., [3, 4]).

According to the Diagnostic and Statistical Manual of Mental Disorders (DSM-5; [5]), the 12-month prevalence of PTSD among North American adults is about 3.5%, while in European, Asian, African, and Latin American countries this has been found to range between 0.5%–1.0%. The World Health Organization World Mental Health Survey showed a 12-month prevalence of 1.1% [6], but these rates were found to vary significantly across adults from low/lower-middle (0.8%), upper-middle (0.7%), and high (1.5%) income countries. A review of the first methodologically sound studies appearing in the 80’s and early 90’s on exposure to traumatic events and PTSD among children and adolescents reported that up to 36% of trauma-exposed children may develop PTSD [7]. The rates of those developing PTSD could be lower in community samples [8] and higher among children experiencing specific human-induced or natural disasters [9]. A meta-analysis of studies published from 1998 to 2011 showed that 15.9% of children and adolescents exposed to a traumatic event developed PTSD [10]. Considering longitudinal data, such as a study by Copeland and his colleagues [11], it was observed that 0.5% of children met the criteria for full-blown PTSD after a traumatic event, while 13.4% developed some PTSD symptoms. A recent systematic review showed that estimates of PTSD are considerably higher among children and adolescents living in low-and middle-income countries (LMICs) than among those living in high-income countries [12], ranging from as low as 0.2% to as high as 87%.

Accumulated epidemiological research has also highlighted different factors associated with the development and maintenance of PTSD [13], which mainly fall into three categories: pre-traumatic (e.g., race/ethnicity, prior psychopathology), peri-traumatic (e.g., duration and severity of trauma experience), and post-traumatic (e.g., access to needed resources, social support). Summarizing studies published up to 2009 on trauma-exposed children and adolescents, a meta-analysis found medium to large effect sizes for many factors related to subjective experience of traumatic events and post-trauma variables, such as low social support, peri-trauma fear, perceived life threat, social withdrawal, comorbid mental health problems, poor family functioning, distraction, and thought suppression [14]. The current literature on predictors of PTSD in trauma-exposed children and adolescents also suggests the involvement of multiple and dynamically linked factors, which impact on children through complex mechanisms. For example, a child’s characteristics like female gender, minority ethnic status, intellectual functioning, comorbid mental health (internalizing, externalizing or psychotic) symptoms, and victimization; as well as family characteristics, like socioeconomic disadvantage, living with one parent, and family history of mental illness, were found the most relevant predictors in a representative cohort of young people in England and Wales [15]. In particular, younger age and female gender are considered strong risk factors for developing PTSD after a traumatic event, but little is known to which extent these contribute together with a type of traumatic events and experiencing specific PTSD symptoms to the presence of PTSD [11, 14], but completely unexplored in adolescents from LMICs.

Current epidemiological data related to mental health problems is urgently required for generating accurate burden estimates and for informing on efficient and appropriate allocation of health resources in LMICs. However, the global coverage of prevalence data for mental disorders in children and adolescents is still limited, with a significant underrepresentation of LMICs [16]. For PTSD, the literature indicates not only on low availability of prevalence studies [17, 18], but also significant variability in PTSD rates in youth across LMICs [12]. Besides, there could be cross-cultural, regional or other contextual differences reflecting significant variability in PTSD rates. Such variability could be also due to methodological differences in sampling and measurement issues. Research shows that the rates and types of traumatic events to which individuals may be exposed to varies according to sociodemographic characteristics and country [1], with many LMICs affected by armed conflict, violence and natural disasters; as well as post-conflict and post-disaster impact on communities (e.g., 19). Consequently, the risk of onset and severity of PTSD may differ across cultural groups, which has led to the introduction of culture-related diagnostic criteria for PTSD [5]. These diagnostic criteria were modified in the DSM-5 [5], which may not necessarily affect the overall PTSD prevalence rates, but may influence the prevalence of specific PTSD symptom criteria (e.g., 20), what could be especially important for LMICs, due to the reported variability in the expression of PTSD symptoms [12].

Considering the abovementioned, additional epidemiological studies are needed from LMICs in relation to PTSD. As part of a larger project with objectives to evaluate broad aspects of adolescent psychopathology under the auspices of the International Child Mental Health Study Group (ICMH-SG, for details see https://www.icmhsg.org/index.php/projects/, [21, 22]), the aim of this study was, therefore, to evaluate the types of traumatic events experienced and the presence and predictors of PTSD symptoms among adolescents from the general population in ten LMICs; Brazil, Bulgaria, Croatia, Indonesia, Montenegro, Nigeria, the Palestinian Territories, the Philippines, Romania, and Serbia. Portugal was included in this study as a reference, high-income country, against which the rates of PTSD symptoms from sampled LMICs were contrasted.

## Methods

The data for this study were simultaneously collected across the countries included. These countries constitute a research network of LMICs available for recruiting participants for various projects (https://www.icmhsg.org/index.php/projects/, [21]). The participants of this study represented a sample of convenience from rural and urban communities across the countries. The same procedures and protocols were followed in setting of the study, recruiting, and testing participants. First, research ethics approval was obtained in each country from the appropriate local research governance or other authorities, and/or research ethics committees. Second, the responsible researcher for each country defined a local, political or administrative zone within a defined included location, with rural and urban communities represented equally. Third, samples from each location were drawn from at least five secondary or equivalent schools, within a frame of randomly selected schools (i.e., simple random sampling). It was estimated that valid data from at least 250 participants were needed for the whole project, but all researchers from each country in the project were encouraged to include more participants if financially and technically possible. Fourth, adolescents were approached by school psychologists/counselors/teachers and were informed about the study. Afterward, from those who agreed to be contacted further, written self and/or parental consent (depending on age) was sought. Fifth, adolescents who provided informed consent, completed the instruments at school to prevent a low response rate. In order to assure anonymity, sealable envelopes were provided to adolescents, in which completed instruments were returned to project assistants.

Inclusion criteria for the present study were age 12–18 years, regular school attendance, reporting at least one traumatic event experienced in the year preceding data collection, and providing written informed consent. Exclusion criteria were inability to read and write, receiving special need assistance, being on an individual learning plan, and having intellectual disability, which were all assessed based on the school records. Since for the present study data were analyzed from a subsample of adolescents experiencing traumatic events, adolescents reporting a medical diagnosis or using substances, or suffering from a functional impairment (e.g., social, occupational) based on school records, were also excluded, since these ones could have disturbances not attributed to PTSD (i.e., H criteria according to the DSM-5; [5]).

### Assessment of traumatic events and PTSD symptoms

#### UCLA PTSD Reaction Index for DSM-5 (PTSD-RI-5)

The UCLA PTSD Reaction Index is a measure of trauma exposure and PTSD symptoms among children and adolescents. The instrument has been widely used over the past two decades and it was revised according to the DSM-5 (PTSD-RI-5; [23]). The self-report version was used in this study and its psychometric properties were reported in a previous study [21]. The instrument has sound psychometric characteristics in the countries included and can be used for in-country assessments of levels and presentations of PTSD, but it cannot be used for cross-cultural comparisons due to insufficient levels of measurement invariance [21]. The first part of the instrument constitutes of 14 plus one optional lifetime trauma screening items (i.e., A criterion for a DSM-5 PTSD diagnosis). The research ethics committees that approved the study in Nigeria and the Palestinian Territories (the shorten term “Palestine” is used in the rest of the text) considered the items “forced to have sex” and “touched private parts without permission” as possibly offending or humiliating to adolescents, thus these were omitted from the instrument. Anyway, participants could mark the item “other traumatic event” and/or write what had happened, thus experiencing sexual assault might have been recorded in their answers. The second part of the instrument consists of 27 items representing specific PTSD symptoms experienced in the previous month, by using a 5-point Likert scale (0 = none of the time, 1 = little, 2 = some, 3 = much and 4 = most of the time). A symptom is considered *present* if the recorded response was ≥ 3 on all items, except items 4, 10 and 46, for which it was ≥ 2. From these items, 20 scores map directly onto the DSM-5 symptom criteria, namely B (i.e., intrusion symptoms), C (i.e., persistent avoidance), D (i.e., negative alterations in cognitions and mood), and E (i.e., alterations in arousal and reactivity), while two into dissociative symptoms (i.e., a dissociative subtype). Severity scores for four subcategories of the PTSD-RI-5, namely B (possible range 0–20), C (possible range 0–8), D (possible range 0–28), and E (possible range 0–24) were calculated as summated scores of all answered items in each scale; where a higher score indicates greater severity. We also calculated a summative score for dissociative symptoms (possible range 0–16). According to the DSM-5 [5], adolescents who had at least one symptom from B, at least one from C, two or more from D, and two or more from E criterion were considered as having a likely diagnosis of PTSD (i.e., full PTSD). Adolescents having PTSD symptoms from one to three criteria were considered as having post-traumatic stress symptoms (PTSS).

### Statistical analysis

Means (M) and standard deviations (SD) for PTSD-RI-5 scores, as well as percentages of specific PTSD symptoms for each DSM-5 criterion, were calculated for each country. Analysis of variance (ANOVA) and χ^2^ were used to test differences in age and gender, respectively. A whole group analysis was performed with adolescents having PTSS vs. full PTSD, excluding Portugal. In this analysis, a binomial logistic regression was performed to ascertain the effects of age, gender, types and numbers of traumatic events, and severity of PTSD symptoms on the probability that participants would have full PTSD rather than PTSS. The amount of missing data ranged between 0.5 and 5% across the countries, and missing data were handled by pairwise deletion. We accepted p < 0.05 as statistically significant level.

## Results

Data for the present study were available from 3370 trauma-exposed adolescents (mean age 15.41 [SD = 1.65] years; 1465 (43.5%) males and 1905 (56.5%) females). There were significant differences across the countries in gender (χ^2^ (df) = 69.22 [10], p < 0.01) and age (F (df) = 96.84 [10], p < 0.01; Table 1).

**Table 1:** Distribution of participants by age and gender

Country	N	Age, M (SD)	Male, N (%)	Female, N (%)
Brazil	187	13.23 (1.27)	80 (42.8)	107 (57.2)
Bulgaria	226	14.87 (1.38)	104 (46.0)	122 (54.0)
Croatia	421	16.44 (1.01)	124 (29.5)	297 (70.5)
Indonesia	419	15.37 (1.33)	166 (39.6)	253 (60.4)
Montenegro	222	15.64 (1.44)	98 (44.1)	124 (55.9)
Nigeria	287	14.54 (1.44)	119 (41.5)	168 (58.5)
Palestine	303	14.99 (2.01)	157 (51.8)	146 (48.2)
Philippines	249	16.53 (0.65)	140 (56.2)	109 (43.8)
Portugal	448	15.75 (1.84)	206 (46.0)	242 (54.0)
Romania	307	15.26 (1.49)	151 (49.2)	156 (50.8)
Serbia	301	15.59 (1.48)	120 (39.9)	181 (60.1)
Total	3370	15.41 (1.65)	1465 (43.5)	1905 (56.5)

### Types and a number of traumatic events experienced

In total, 874 (25.9%) adolescents reported one traumatic event experienced in the year preceding data collection, while 771 (22.9%) reported two, and 1725 (51.2%) three or more. The most frequently reported traumatic events were death of a close person (69.7%), witnessing other than domestic violence (40.5%), being in a natural disaster (34.4%) and witnessing violent death or serious injury of a close person (33.9%; Table 2).

**Table 2:** Types of traumatic events experienced

Traumatic event	N (%)
Natural disaster	1158 (34.4)
Serious accident	626 (18.6)
War exposure	495 (14.7)
Direct victim of domestic violence	519 (15.4)
Witnessing domestic violence	643 (19.1)
Other direct violence exposure	667 (19.8)
Witnessing other violence	1362 (40.5)
Facing a dead body	956 (28.4)
Touched private parts without permission	339 (14.4)
Violent death or serious injury of a close person	1140 (33.9)
Exposure to medical treatments/procedures	653 (19.4)
Forced to have sex	108 (4.6)
Death of a close person	2348 (69.7)
Other traumatic event	924 (27.7)

### PTSD symptom distribution

The distribution of specific symptoms across the countries is given in Table 3. In the whole sample, B criterion symptoms were the most frequently present (45%), followed by symptoms of D (33.8%), C (26.9%) and E criterion (22.5%), and dissociative symptoms (20.4%).

**Table 3:** Distribution of specific DSM-5 PTSD symptoms, N (%)

Country	B criterion	C criterion	D criterion	E criterion	Dissociative
Brazil	108 (57.8)	51 (27.3)	76 (40.6)	40 (21.4)	30 (16.0)
Bulgaria	68 (30.1)	55 (24.3)	54 (23.9)	42 (18.6)	44 (19.5)
Croatia	156 (37.1)	105 (24.9)	139 (33.0)	88 (20.9)	54 (13.0)
Indonesia	161 (38.4)	83 (19.8)	97 (23.2)	74 (17.7)	36 (8.6)
Montenegro	82 (36.9)	60 (27.0)	58 (26.1)	38 (17.1)	40 (18.4)
Nigeria	165 (57.5)	99 (34.5)	135 (47.0)	84 (29.3)	125 (45.3)
Palestine	206 (68.0)	123 (40.6)	142 (46.9)	80 (26.4)	76 (25.1)
Philippines	150 (60.2)	66 (26.5)	149 (59.8)	83 (33.3)	130 (52.2)
Portugal	184 (41.1)	149 (33.3)	126 (28.1)	109 (24.3)	64 (14.3)
Romania	142 (46.3)	53 (17.3)	90 (29.3)	60 (19.5)	49 (16.0)
Serbia	93 (30.9)	63 (20.9)	73 (24.3)	61 (20.3)	41 (13.6)
Total	1515 (45)	907 (26.9)	1139 (33.8)	759 (22.5)	689 (20.4)

Symptoms from at least one criterion were present in 689 (20.4%) adolescents, symptoms from two criteria in 537 (15.9%), symptoms from three criteria in 423 (12.6%), and symptoms from all four criteria (i.e., full PTSD), in 322 adolescents (9.6%; Table 4). The rates of adolescents with a likely PTSD diagnosis were lowest in Indonesia (6.2%) and highest in Nigeria (15.3%). All adolescents with symptoms from four criteria also had dissociative symptoms.

**Table 4:** Distribution of the number of PTSD symptoms from the DSM-5 B, C, D, and E criteria

Country	No symptoms		Symptoms from one criterion		Symptoms from two criteria		Symptoms from three criteria		Symptoms from four criteria	
	N	%	N	%	N	%	N	%	N	%
Brazil	55	29.4	49	26.2	41	21.9	24	12.8	18	9.6
Bulgaria	119	52.7	43	19.0	32	14.2	16	7.1	16	7.1
Croatia	206	48.9	78	18.5	45	10.7	48	11.4	44	10.5
Indonesia	214	51.1	89	21.2	48	11.5	42	10.0	26	6.2
Montenegro	110	49.5	48	21.6	20	9.0	26	11.7	18	8.1
Nigeria	90	31.4	44	15.3	64	22.3	45	15.7	44	15.3
Palestine	68	22.4	65	21.5	64	21.1	66	21.8	40	13.2
Philippines	69	27.7	21	8.4	73	29.3	63	25.3	23	9.2
Portugal	184	41.1	100	22.3	72	16.1	44	9.8	48	10.7
Romania	120	39.1	100	32.6	41	13.4	21	6.8	25	8.1
Serbia	164	54.5	52	17.3	37	12.3	28	9.3	20	6.6
Total	1399	41.5	689	20.4	537	15.9	423	12.6	322	9.6

### Predictors of having full PTSD vs. PTSS

Table 5 shows the results of the logistic regression analysis. The logistic regression model created was significant (χ^2^ = 583.54, p < 0.01), with 73.5% (Nagelkerke R^2^) of the variance explained and 94.2% of cases correctly classified. In the model, age, six different traumatic events (i.e., natural disaster, serious accident, war exposure, witnessing domestic violence, facing a dead body, and forced to have sex), and severity symptoms of C, D, and E criterion, emerged as predictors.

**Table 5:** Logistic regression results

Predictors^a^	B	SE	Wald (df)	p value	Exp(B) (95% CI)
Age	−0.24	0.10	5.46 (1)	0.02	0.79 (0.65–0.96)
Gender (female)	0.29	0.32	0.83 (1)	0.36	1.34 (0.71–2.53)
Natural disaster	−0.85	0.35	5.99 (1)	0.01	0.42 (0.21–0.84)
Serious accident	−1.11	0.42	6.82 (1)	0.01	0.32 (0.14–0.75)
War exposure	1.12	0.46	5.91 (1)	0.02	3.06 (1.24–7.56)
Direct victim of domestic violence	−0.69	0.44	2.49 (1)	0.11	0.49 (0.21–1.18)
Witnessing domestic violence	−0.92	0.42	4.80 (1)	0.03	0.39 (0.17–0.90)
Other direct violence exposure	−0.08	0.38	0.04 (1)	0.83	0.92 (0.43–1.97)
Witnessing other violence	−0.27	0.34	0.62 (1)	0.42	0.75 (0.38–1.50)
Facing a dead body	−1.17	0.34	11.44 (1)	< 0.01	0.30 (0.15–0.61)
Touched private parts without permission	−0.77	0.40	3.65 (1)	0.06	0.46 (0.20–1.01)
Violent death or serious injury of a close person	0.02	0.32	0.01 (1)	0.94	1.02 (0.54–1.92)
Exposure to medical treatments/procedures	0.41	0.36	1.31 (1)	0.25	1.51 (0.74–3.08)
Forced to have sex	1.22	0.55	4.85 (1)	0.03	3.39 (1.14–10.04)
Death of a close person	0.14	0.36	0.15 (1)	0.69	1.15 (0.56–2.32)
Other traumatic event	0.23	0.35	0.42 (1)	0.51	1.25 (0.63–2.50)
One traumatic event	–	–	1.36 (2)	0.51	–
Two traumatic events	−0.21	0.56	0.15 (1)	0.69	. 80 (0.26–2.41)
Three or more traumatic events	0.34	0.64	0.27 (1)	0.59	1.40 (0.39–4.98)
B symptoms severity	0.04	0.04	0.86 (1)	0.35	1.04 (0.95–1.13)
C symptoms severity	1.02	0.10	87.91 (1)	< 0.01	2.79 (2.25–3.46)
D symptoms severity	0.12	0.04	8.66 (1)	< 0.01	1.13 (1.04–1.23)
E symptoms severity	0.47	0.05	77.14 (1)	< 0.01	1.60 (1.44–1.77)
Dissociative symptoms severity	−0.07	0.04	2.81 (1)	0.09	0.92 (0.84–1.01)

^a^Dependent variable was full PTSD vs. PTSS

Younger adolescents were 0.79 times more likely to have full PTSD than PTSS. Adolescents reporting to have been exposed to war and forced to have sex were 3.06 and 3.39 times respectively more likely to have full PTSD than PTSS. Adolescents with more severe symptoms from C, D, and E criteria had 2.79, 1.13, and 1.60 times respectively greater chance to have full PTSD. However, adolescents who reported experiencing a natural disaster, serious accident, witnessing domestic violence or facing a dead body as a traumatic event were 0.3–0.42 times more likely to have PTSS than full PTSD.

## Discussion

The highest rates for traumatic events reported in our study were being exposed to death of a close person, witnessing violence other than domestic, experiencing a natural disaster, and witnessing violent death or serious injury of a close person; while much lower rates were reported for the other events. These rates of exposure to different traumatic events closely corresponded to those detected in previous studies (e.g., [2, 10, 17, 24]). Of particular importance are two findings related to the type and number of traumatic events experienced. First, some form of sexual assault was reported by 14.4% of adolescents in the whole sample, while 4.6% reported to have been victims of forced sex. Approximately 15% of adolescents in the general population in high-income countries reported at least one sexual assault (e.g., [2, 25]) and a similar rate of 12% was found in some East and Southern Africa countries [26]. In addition, 14.7% of adolescents reported to have been exposed to war conflict, which is a significant proportion. The other important finding is that every second adolescent reported to have had experienced three or more traumatic events, which is substantially high and exceeds previously reported rates [2].

Turning to the findings related to the presence of DSM-5 symptoms, B criteria (i.e., intrusions) were the most frequently present, followed by D (i.e., negative alterations in cognitions and mood), C (i.e., persistent avoidance), E (i.e., alterations in arousal and reactivity), and lastly dissociative symptoms. Although symptoms from B criterion were the most frequently present in each country, followed by symptoms of D criterion, the distribution of specific PTSD symptoms varied from low to high across the countries. In overall, nearly every forth adolescent had PTSD symptoms from two or three DSM-5 criteria, which may correspond to partial/subsyndromal PTSD [20, 27], while 9.6% of the whole sample met all four criteria (i.e., likely having DSM-5 diagnosis of PTSD), specifically 9.1% if excluding Portugal. Focusing only on those who may have full PTSD, the percentage range was 6.2–8.1% for adolescents from Indonesia, Serbia, Bulgaria, and Montenegro and 9.2–10.5% for adolescents from the Philippines, Croatia, and Brazil. From Portugal, 10.7% adolescents fall into this criterion, while 13.2% and 15.3% for Palestine and Nigeria, respectively. However, the distribution of adolescents fulfilling two to four DSM-5 criteria varied also substantially across the countries. In addition, dissociative symptoms were also frequent and varied across the countries. However, dissociative symptoms are in general highly prevalent among youth receiving psychiatric care and are often linked to the exposure of various types of traumas [28].

Compared to the data from a meta-analysis, which showed that the pooled prevalence rate of children and adolescents exposed to a traumatic event was 15.9% (95% CI 11.5–21.5; 10), adolescents in our sample, except those living in Palestine and Nigeria, had lower rates of PTSD. In this regard, adolescents in LMICs may not necessarily have higher levels of PTSD as compared to adolescents living in high-income countries. However, substantial variability in developing PTSD across countries has previously been noted [12], which is consistent with the pattern of our findings. In general, our finding implies that adolescents from different regions/countries may have different propensity to developing and manifesting PTSD symptomatology. The observed variability may be explained by specific regional/country moderators and mediators operating with known factors associated with the development and maintenance of PTSD [13]. For example, the variability may be explained by collective events such as war trauma or natural disasters affecting specific regions and populations, but not the majority who are more likely to be exposed to chronic life events associated with disadvantage. Another of social-emotional processes [29], which may lead to PTSD symptoms. For example, both cultural differences in relation to emotional dysregulation and associated PTSD symptoms [30], as well as in neural correlates of affective and cognitive functions that impact on the subsequent manifestation and progression of PTSD [31], have been found.

There are also some differences comparing our data to previous studies with adolescents from the countries included here. For example, PTSD rates found in different Nigerian regions ranged between 2.4 and 9.9% [24, 32], while in Brazilian regions between 2.3 and 12.4% [33, 34]. In an Indonesian sample, 22.4% of adolescents had PTSD symptoms after a tsunami [35]. Data related to war and armed conflict showed that 57% of adolescents in Uganda had PTSD [36], 13.7% of refugee children and adolescents had PTSD symptoms in Croatia [37]. Prevalence rates among all children and adolescents were estimated to be between 5 and 8% in Israel, 23–70% in Palestine and 10–30% in Iraq [38]. There are several possible explanations for the observed differences between our study and previous research. First, we considered the rates of developing PTSD, irrespective of the type of traumatic events, whereas a great majority reported experiencing two or more events. Second, the DSM-5 criteria were considered in our sample, while all previous studies mostly used the DSM-IV criteria. Third, there might be different risk factors operating in the development of PTSD across countries, with some adolescents from some countries being particularly vulnerable to PTSD symptoms. Of particular importance here are collective events such as war trauma, armed conflicts or natural disasters collectively affecting included regions and populations. Fourth, the differences may be due to using different instruments [35] or semi-structured and diagnostic interviews [32, 33].

Finally, we evaluated possible predictors for having full PTSD compared to PTSS. The results indicate that younger adolescents were at a slightly greater risk for having full PTSD than PTSS. Two previous studies also reported that younger age is a risk factor for developing PTSD (e.g., [13, 39]). On one hand, age could be considered as a specific pre-trauma risk for PTSD, but on the other, there may be a number of different age-dependent vulnerabilities involved in the development to PTSD, such as lower ability of younger to recover from or adjust to a traumatic event [40], lower levels of posttraumatic growth [41], greater impact of traumatic events [42] or more specific cognitive vulnerabilities to PTSD [43, 44]. Furthermore, our results showed that there are some differences related to the type of traumatic events and experiencing PTSD, regarding what was previously observed. For example, a meta-analysis reported that following a non-interpersonal trauma, PTSD rates could be 9.7% (95% CI 6.1–15.2), whereas following interpersonal trauma these would rise to 25.2% (95% CI 16.8–35.8; [10]). In our sample, adolescents who reported to have been exposed to war and forced to have sex were about three times more likely to have full PTSD than just PTSS, but the probability of having full PTSD could be up to seven times higher for experiencing war and ten times higher for experiencing forced sex. Exposure to war trauma and sexual violence were previously noted as the traumatic events most frequently associated with PTSD (e.g., [45, 46]). In our sample, adolescents who experienced a natural disaster, serious accident, witnessing domestic violence or facing a dead body were at greater risk of fulfilling PTSS than full PTSD criteria, while other traumatic events did not emerge as PTSD predictors. In addition, the likelihood of having full PTSD was associated with more severe avoidance, negative alterations in cognitions and mood, and alterations in arousal and reactivity. The probability was especially high for the severity of C criterion symptoms, which indicates that adolescents showing higher levels of avoidance of trauma-related stimuli after the trauma are more likely to have full PTSD. This last finding is in line with previous research in indicating that greater avoidance predicts more severe PTSD symptoms (e.g., [47]) and with DSM-5 criteria, which distinguished these criteria from other symptoms [5]. Previous studies showed that maladaptive cognitive appraisals play an important role in the development and persistence of PTSD in children and adolescents [48–50]. The severity of B criterion symptoms was not predictive of having full PTSD vs. only PTSS in our analysis, which could imply that it is more relevant the presence of intrusion symptoms (i.e., a categorical aspect) rather than how severe these are for the final outcome (i.e., a dimensional aspect). Taken together, intrusions if present would lead to either PTSS or PTSD diagnosis, as the presence of other criteria symptoms, but it is the level of intensity of the other symptoms and not B criterion, which will predict the development of PTSS or full PTSD.

The study has several strengths. We included an adolescent sample only, which allowed us to estimate clearer PTSD rates, rather than including mixed samples of children and adolescents. Adolescents from ten LMICs and one high-income country were included. For some countries, like Serbia, Bulgaria, or Romania, this was the first time that rates for PTSD were reported, which is important for policy development, service planning and staff training [16]. In addition, the scale used, PTSD-RI-5, allowed us to assess specific traumatic events and to estimate the rates based on the latest DSM-5 criteria, ensuring that strict psychometric properties were followed. However, there are also some limitations that need to be acknowledged. First, there may be an imbalance between the countries and the continents included to generalize the findings. Also, the countries included were not selected following specific criteria for ensuring representativeness of economic, political, or religious aspects or previously known rates of traumatic events, like war or human and natural disasters. For example, we included only one high-income country, Portugal, as a reference point, which is among countries with lower incomes in the European Union and does not necessarily represent all high-income countries. Second, participants were sampled from regions of convenience and the sample size varied across countries, thus it is possible that the same levels of variability in expressions of PTSD symptoms were not captured, which also limits the generalizability of the results. Third, even though the PTSD-RI-5 has been shown to have robust psychometric properties [21], by only using its self-report version without adult corroboration, youth may have over- or, more likely, under-reported PTSD symptoms. Fourth, besides self-report and school records, no other means were considered to assess youth mental and physical health and to confirm the reported medical diagnoses. In addition, using substances was not assessed separately. Fifth, using a structured diagnostic interview with those who screened positive for symptoms would have estimated more accurate prevalence rates of PTSD.

## Conclusions

This study showed that almost every third adolescent from the general population of LMICs might be suffering from some PTSD symptoms after experiencing a traumatic event, while about 9% might have sufficient symptoms to be diagnosed with the DSM-5 PTSD. Although, these rates may not be necessary higher than in high-income countries, the distribution of PTSD symptoms and their severity levels varied markedly across LMICs countries. Younger adolescents, those exposed to war and forced to have sex, and those with more severe PTSD symptoms, especially avoidance, are at a greater risk for having full PTSD.

There are some implications for practice and services from our results. First, it is important that health and social care services work collaboratively, especially in relation to child protection, in developing joint policies training and care pathways. Second, the results may help in planning and generating specific interventions for LMICs, such as a stepped care approach, considering the level of resources, involving community volunteers and other non-specialists, and contextualizing interventions such as narrative exposure therapy to different sociocultural contexts [18, 51]. Third, our data indicate needs to further study cultural differences in PTSD propensity across multiple countries/regions, as well as to study different cultural models with multiple predictors, because there might be collective exposure to trauma such as natural disasters or war conflict, or in association with extreme socioeconomic disadvantage, which could explain the differences. Fourth, the observed variability in the presence of symptoms and traumatic events needs to be considered by combining cross-cultural and neurobiological perspectives of PTSD. These connectivity differences, in turn, maybe associated with higher cortisol levels in anticipation of social stress. Early childhood poverty may relate to alterations in resting brain function, as well as greater peripheral stress reactivity. Finally, our results may help in generating PTSD burden estimates, and informing allocation of health resources in LMICs. Additional epidemiological research is needed, considering the above limitations, in order to reach more accurate and precise decisions.

## Data Availability

The datasets used and analyzed during the current study are available from the corresponding author on reasonable request.

## Abbreviations

DSM-5: Diagnostic and statistical manual of mental disorders (DSM-5)
ICMH—SG: International Child Mental Health Study Group
LMICs: Low- and middle-income countries
PTSD: Posttraumatic stress disorder
PTSS: Post-traumatic stress symptoms
UCLA PTSD Index: The University of California, Los Angeles, Post-traumatic Stress Disorder Reaction Index
PTSD-RI-5: UCLA PTSD Reaction Index for DSM-5 (PTSD-RI-5
